# Field Testing of a Controlled-Source Wide Frequency Range Magnetotelluric Detector Using SQUID and Inductive Magnetic Sensors

**DOI:** 10.3390/s25133896

**Published:** 2025-06-23

**Authors:** Zucan Lin, Qisheng Zhang, Rongbo Zhang, Xiyuan Zhang, Hui Zhang, Xinchang Wang, Huiying Li, Yunheng Liu, Bojian Zhou, Jian Shao, Keyu Zhou

**Affiliations:** School of Geophysics and Information Technology, China University of Geosciences (Beijing), Beijing 100083, China; 3010220022@email.cugb.edu.cn (Z.L.); 2110220014@email.cugb.edu.cn (H.L.); keyuzhou@email.cugb.edu.cn (K.Z.)

**Keywords:** geophysical instruments, Controlled-Source electromagnetic method, wide-band receiver, SQUID magnetic sensor, inductive magnetic sensor, field experiments

## Abstract

To enhance the resolution of shallow geological structure detection, this study developed a Controlled-Source wide frequency range Magnetotelluric Detector (called CSUMT) with a frequency range spanning from 1 Hz to 1 MHz, and conducted systematic field experiments in Fengxian County, Shaanxi Province. The CSUMT system employs a high-precision 24-bit analog-to-digital converter and is compatible with both inductive magnetic sensors and superconducting quantum interference device (SQUID) magnetic sensors, featuring wide bandwidth and high dynamic range. Comparative experiments with the commercial V8 instrument demonstrated high consistency in electric field, magnetic field, and apparent resistivity measurements, confirming the CSUMT system’s reliability in field applications. In addition, this study compared the performance of inductive and SQUID magnetic sensors in actual surveys, revealing that SQUID sensors exhibit lower noise and more stable data output, making them suitable for signal detection across a broader frequency range. The results validate the practicality of the CSUMT system in complex geological environments and provide experimental support for the appropriate selection of magnetic sensors.

## 1. Introduction

Magnetotellurics (MT) is a geophysical method that utilizes natural electromagnetic fields to investigate the electrical structure of the subsurface. In 1950, Tikhonov proposed a theoretical approach for determining the electrical properties of the deep layers of the Earth’s crust based on the relationships between magnetic and electric fields in the natural electromagnetic field of the Earth as derived from Maxwell’s equations [1]. In 1953, Cagniard put forward the basic theory of the magnetotelluric method of geophysical prospecting [2]. MT employs magnetic sensors and grounded electrodes to measure the amplitudes of orthogonal components of the magnetic and electric fields, as well as the phase of their impedance. The apparent resistivity is then calculated using the following formula:

(1) $$\rho_{\alpha }=\frac{1}{\mu \omega }\frac{{\left|E\right|}^{2}}{{\left|H\right|}^{2}}$$

This value reflects the resistivity characteristics of the subsurface media at specific depths. In Equation (1), *E* represents the intensity of the electric field, *H* represents the intensity of the magnetic field, and μ represents the magnetic permeability of the medium, while ω denotes the angular frequency of the electromagnetic wave.

**Figure 1:**
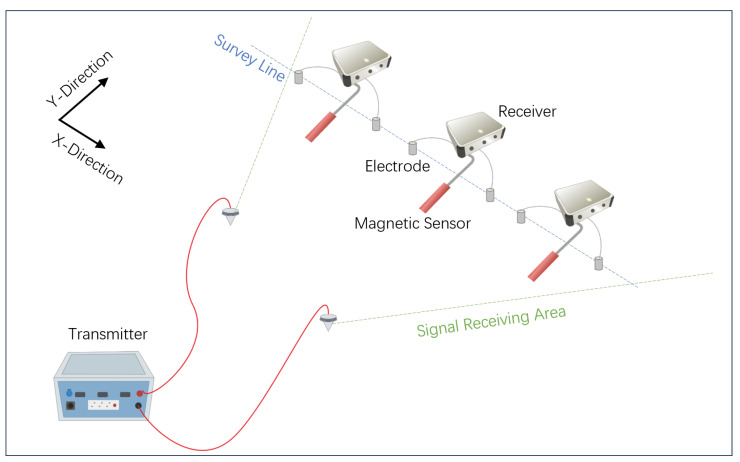
Schematic diagram of the Controlled-Source magnetotelluric measurement setup.

Magnetotellurics suffers from limitations such as unstable natural sources and low signal-to-noise ratios. Controlled-Source magnetotellurics (CSMT) is a method that utilizes artificially generated electromagnetic fields deployed on the surface to replace natural sources [3]. It measures the subsurface media’s response to these sources to infer the underground electrical structure [4]. A schematic diagram of the CSMT setup is shown in Figure 1.

Compared to natural-source MT, CSMT offers advantages such as higher signal-to-noise ratio and stronger resistance to interference [5]. It has been widely applied in resource exploration, environmental monitoring, and other fields [6,7,8]. Currently, several companies have developed mature CSMT instruments, including the V8 Networked Multifunction Receiver by Phoenix Geophysics, the Stratagem EH-5 by Geometrics, and the GDP32II by Zonge International, Inc. Their key specifications are listed in Table 1 [9,10,11].

As shown in Table 1, modern commercial electromagnetic exploration systems are generally characterized by multi-channel support, wide bandwidth, high-resolution analog-to-digital conversion, broad dynamic range, compact size, and lightweight design. These features make them well-suited for detecting weak electromagnetic signals under complex field conditions.

However, traditional CSMT systems typically operate below 100 kHz. This is because electromagnetic fields attenuate more rapidly underground at frequencies above 100 kHz, and the effects of displacement currents become more significant. Nevertheless, higher operating frequencies can improve resolution for shallow subsurface investigations, which are often required in near-surface engineering applications.

Bastani [12,13] and Saraev [14,15] conducted CSMT surveys aimed at extending the working frequency toward the high-frequency end. Bastani’s system achieved signal acquisition up to 250 kHz, while Saraev considered the design of a CSRMT transmitter with an operating frequency up to 1000 kHz. However, in these earlier studies, the effective transmitted frequencies often did not fully cover the high end of the range, and measurements at higher frequencies sometimes relied on harmonic components of lower-frequency sources. This approach led to limitations such as reduced signal-to-noise ratios and difficulty in achieving stable and accurate high-frequency responses.

To address these limitations, our work significantly increases the transmitter’s primary frequency, enabling direct measurements at the target frequency range rather than depending on harmonics. This approach enhances the signal-to-noise ratio and improves data quality in high-frequency surveys. Moreover, our Controlled-Source wide frequency range Magnetotelluric Detector (called CSUMT) extends the frequency range even further, from 1 Hz to 1 MHz, providing a broader frequency band to better resolve subsurface electrical structures in complex geological environments.

To accommodate the wideband electromagnetic signal acquisition requirements, this study adopts a specially designed wideband magnetic sensor. Magnetic sensors are key components of CSMT systems, and the main types include inductive magnetic sensors [16], fluxgate magnetometers [17], and superconducting quantum interference device (SQUID) magnetic sensors [18]. The non-magnetic dewar of the SQUID magnetic sensor has been optimized to a diameter of 308 mm and a height of 550 mm, making it suitable for transport and use in challenging field terrains. A key advantage of the SQUID magnetic sensor is its ability to accurately determine the direction of the magnetic field vector with high spatial resolution (on the order of millimeters), whereas conventional magnetic sensors may exhibit significant uncertainties in vector orientation due to lower spatial resolution.

Inductive magnetic sensors are widely used due to their high sensitivity and low noise levels; however, their sensitivity declines rapidly at low frequencies, making them less suitable for ultra-low frequency magnetic field measurements. Major manufacturers of inductive magnetic sensors include Phoenix Geophysics (Canada) and Metronix (Germany).

Fluxgate magnetometers offer stable sensitivity across frequencies and can compensate for the low-frequency limitations of inductive sensors. Key producers include Bartington (UK) and Magson (Germany).

In this study, we use a high-frequency-optimized inductive magnetic sensor developed by the Institute of Geology and Geophysics, Chinese Academy of Sciences. It features a flat response band from 900 Hz to 750 kHz. Its noise data is 1.6 $\mathrm{pT}/\sqrt{\mathrm{Hz}}$ at 20 Hz, 1.9 $\mathrm{fT}/\sqrt{\mathrm{Hz}}$ at 100 kHz, and 3.2 $\mathrm{fT}/\sqrt{\mathrm{Hz}}$ at 500 kHz.

SQUID magnetic sensors provide constant sensitivity across the full frequency spectrum and exhibit extremely low noise levels, but practical implementation challenges remain. Supracon AG (Germany) leads internationally in commercialized SQUID magnetic sensors, while the Shanghai Institute of Microsystem and Information Technology, Chinese Academy of Sciences, has also independently developed its own SQUID technology.

In this study, we employ a SQUID magnetic sensor developed by the Shanghai Institute of Microsystem and Information Technology, with a bandwidth from DC to 1 MHz. Its noise data is 3.75 $\mathrm{fT}/\sqrt{\mathrm{Hz}}$ at 20 Hz, 2.45 $\mathrm{fT}/\sqrt{\mathrm{Hz}}$ at 100 kHz, and 2.08 $\mathrm{fT}/\sqrt{\mathrm{Hz}}$ at 500 kHz.

To evaluate the reliability and accuracy of the CSUMT detector, a comparative field experiment was conducted in Fengxian County, Shaanxi Province, China, between the CSUMT detector and commercial electromagnetic detection instruments. Both inductive magnetic sensors and SQUID magnetic sensors were used for detection experiment. Their sensitivity, background noise, and environmental adaptability were compared to provide a reference for magnetic sensor selection in CSUMT applications.

## 2. CSUMT Detector

**Figure 2:**
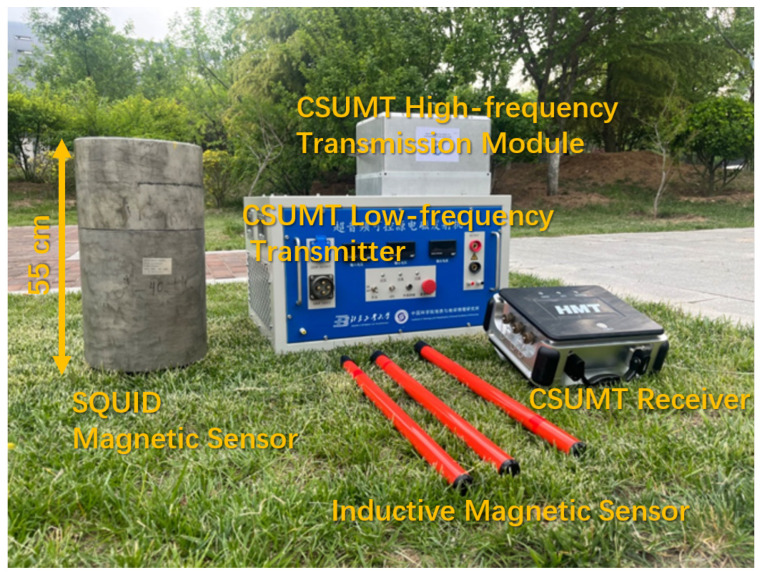
Photograph of the CSUMT detector.

As shown in Figure 2, the CSUMT detector consists of six main components: a low-frequency transmitter, a high-frequency transmission module, a receiver, non-polarizable electrodes, inductive magnetic sensors, and a SQUID magnetic sensor. The CSUMT detector is suitable for both MT and CSMT methods.

The main specifications of the CSUMT receiver are listed in Table 1. It employs a 24-bit high-speed, high-precision Sigma-Delta (Σ−Δ) analog-to-digital converter with a maximum sampling rate of 2.5 MSPS. The receiver integrates a cascaded digital filtering and down-sampling IP core, extending the minimum sampling rate to 305 SPS, making it suitable for low-frequency detection scenarios. The CSUMT receiver is compatible with both inductive magnetic sensors and SQUID magnetic sensors, which can be selected based on the specific application requirements.

**Figure 3:**
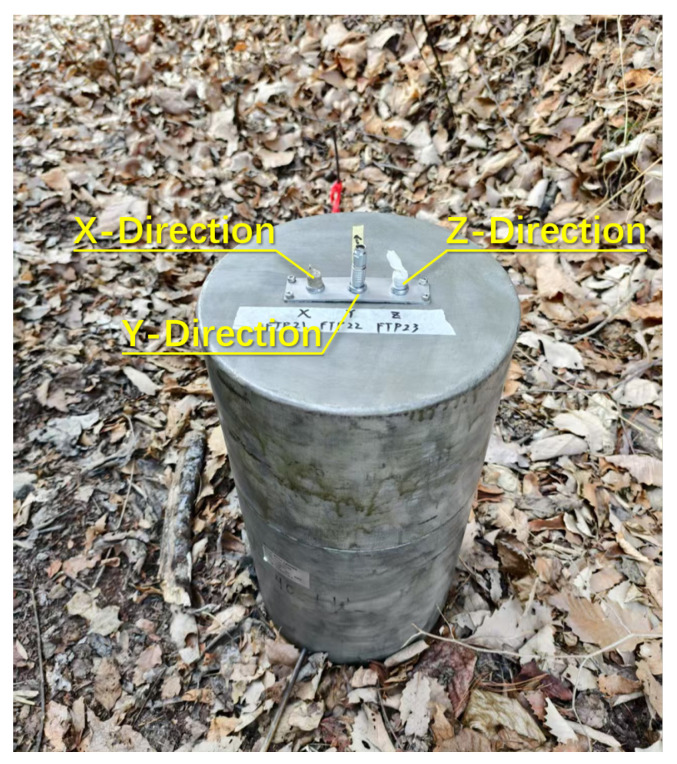
Photograph of the SQUID magnetic sensor.

The physical appearance of the SQUID magnetic sensor is shown in Figure 3. It has a cylindrical shape with three signal output ports on the top surface, corresponding to magnetic field measurements in two horizontal directions and one vertical direction, respectively. For electric field measurements, non-polarizable electrodes are used. These electrodes are buried in the ground during operation to measure the electric potential difference between two points spaced at a fixed distance.

Due to the different transmitter–receiver distances required in low- and high-frequency applications, the CSUMT transmitter system is divided into a low-frequency transmitter and a high-frequency transmission module. The high-frequency module can operate independently or be connected to and powered by the low-frequency transmitter, which also handles its control functions. The low-frequency transmitter operates in the frequency range of 0.125 Hz to 9600 Hz, while the high-frequency transmission module covers the range from 9600 Hz to 1 MHz.

Upon startup, the CSUMT detector automatically transmits and receives electromagnetic signals according to a pre-set frequency schedule stored in both the transmitter and receiver, synchronized with GNSS time. In addition, the CSUMT detector supports manual operation for specific frequencies. Operators can connect to the instrument via a wired or wireless local area network to monitor the transmitted and received signal waveforms in real time.

## 3. Comparison Experiment Conducted in Shaanxi Province

### 3.1. Test Design and Field Work

**Figure 4:**
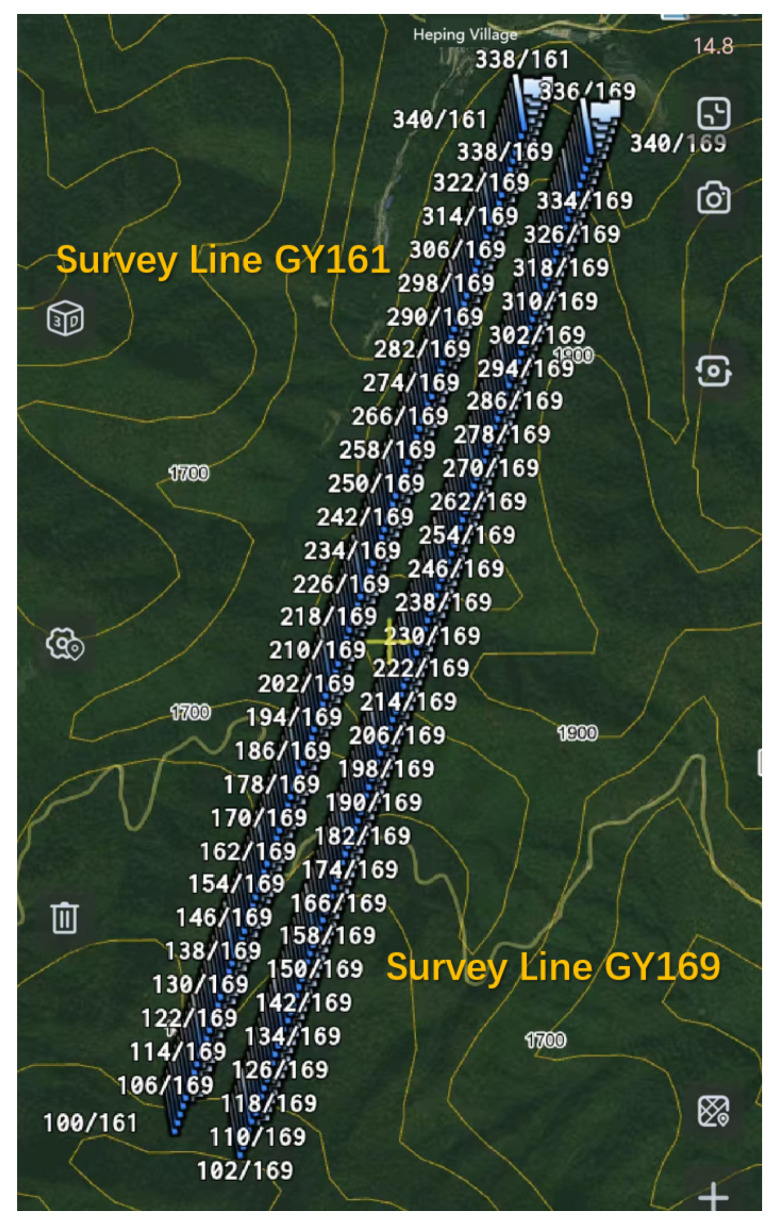
Survey lines in Fengxian County, Shaanxi Province.

To verify the field applicability and measurement accuracy of the CSUMT detector, a comparison experiment was conducted between the CSUMT detector and the V8 receiver in the Erlihe lead–zinc mining area of the Fengxian County ore concentration zone, Shaanxi Province. This area was chosen due to the availability of extensive geological data and previous geophysical surveys. Two survey lines, GY161 and GY169, were laid out for the comparison experiment, with a spacing of 25 m between measurement points, resulting in a total of 240 stations. The geographic locations of the two survey lines are shown in Figure 4. Each line is 3 km in length, with a spacing of 200 m between them and an orientation of 20°.

**Figure 5:**
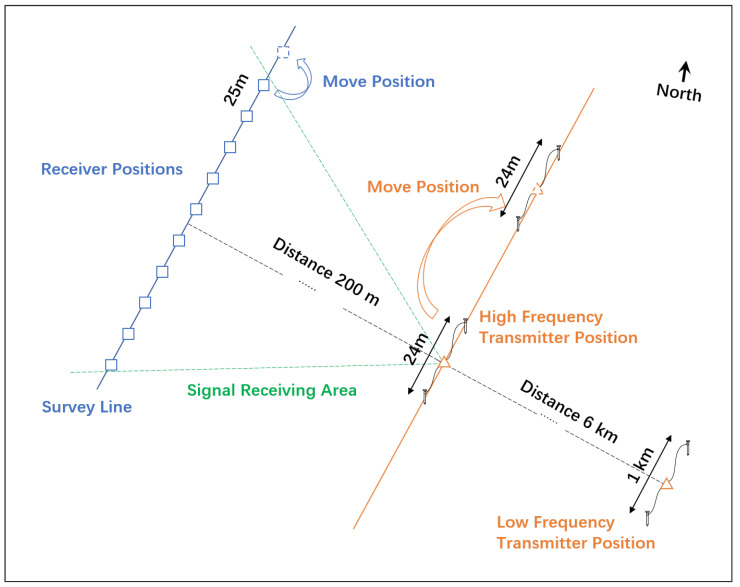
Schematic diagram of the relative positions of transmitter and receivers.

**Figure 6:**
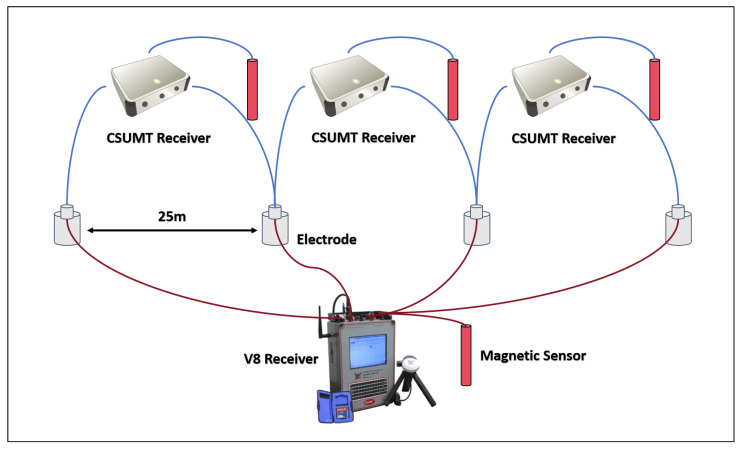
Schematic illustration of the station deployment for CSUMT and V8 receivers.

**Figure 7:**
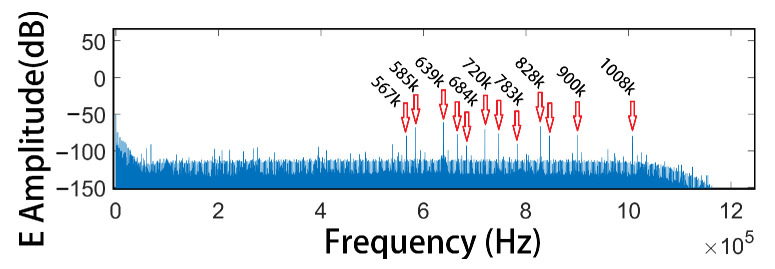
Electric field spectrum received by the receiver when the transmitter is off, showing the spectral lines of various radio stations.

As shown in Figure 5, the low-frequency transmission point is located approximately 6 km from the survey lines, with a transmitter electrode spacing of 1 km. The high-frequency transmission point is about 200 m from the survey lines, with an electrode spacing of 24 m. The high-frequency transmitter moves parallel to the survey line as the receiving points shift. The station layouts for the CSUMT and V8 receivers are illustrated in Figure 6. Measurement stations are arranged in groups of three points, with adjacent stations sharing one electrode. The electric field component along the survey line and the magnetic field component perpendicular to the line are measured. Because the transmitter’s frequency range is sufficiently high, we performed measurements directly at the fundamental frequency of the transmitted signal. The fundamental frequencies used in the experiment and the corresponding acquisition durations are listed in Table 2. One complete acquisition cycle across all frequencies took approximately 25 min. Since the V8 system does not record signals above 10 kHz, no transmission or acquisition was conducted at 10 kHz. Figure 7 shows the spectrum of the electric field signals received by the receiver when the transmitter is off, where the spectral lines of various frequency-modulated radio stations can be observed.

**Figure 8:**
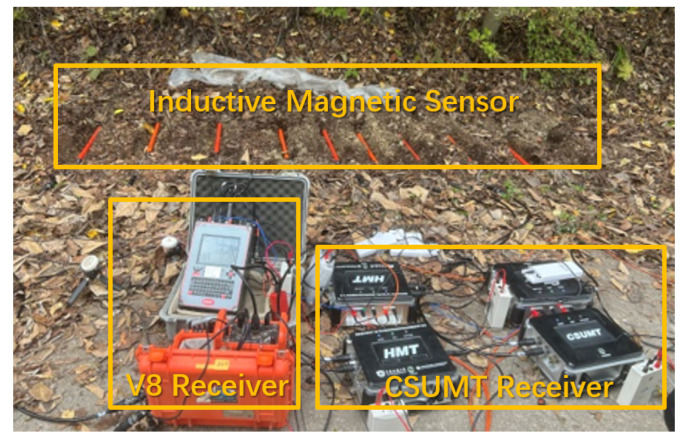
Photograph of the comparison experiment site.

Three CSUMT receivers and one V8 receiver were used to measure each group of stations. At each point, the receivers shared the same set of electrodes, while each receiver used its own magnetic sensor. A photograph of the field setup is shown in Figure 8. Both the CSUMT and V8 receivers simultaneously recorded signals transmitted by the CSUMT transmitter, acquiring electric field, magnetic field, and apparent resistivity data at the corresponding points.

### 3.2. Comparison Experiment Results

**Figure 9:**
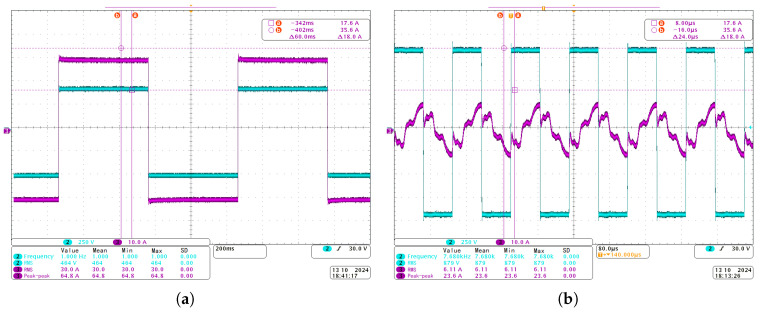
Time-domain voltage and current waveforms of the CSUMT transmitter. (**a**) Voltage and current waveforms for the 1 Hz signal. (**b**) Voltage and current waveforms for the 7.68 kHz signal.

The CSUMT transmitter emits electromagnetic field signals with a frequency range from 1 Hz to 9600 Hz. The voltage and current waveforms are shown in Figure 9, where the light blue curve represents the voltage waveform, and the purple curve represents the current waveform. The root mean square (RMS) value of the voltage for the 1 Hz signal reaches 464 V, while the RMS value of the current is 30 A. Similarly, for the 7.68 kHz signal, the RMS voltage reaches 879 V, and the RMS current is 6.11 A.

**Figure 10:**
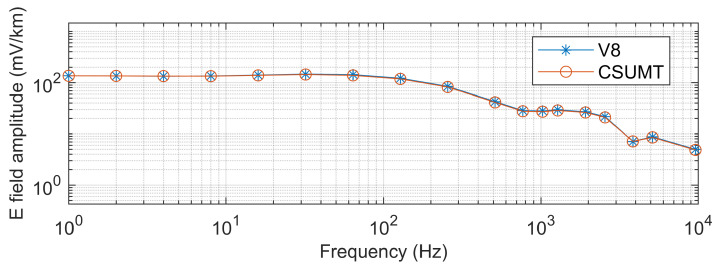
Comparison of electric field measurements between CSUMT and V8 receivers at the 1000 m point of the GY161 survey line.

**Figure 11:**
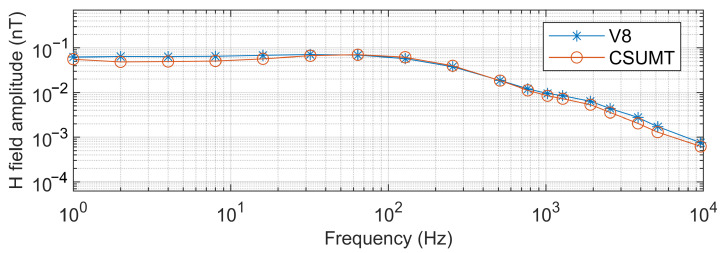
Comparison of magnetic field measurements between CSUMT and V8 receivers at the 1000 m point of the GY161 survey line.

**Figure 12:**
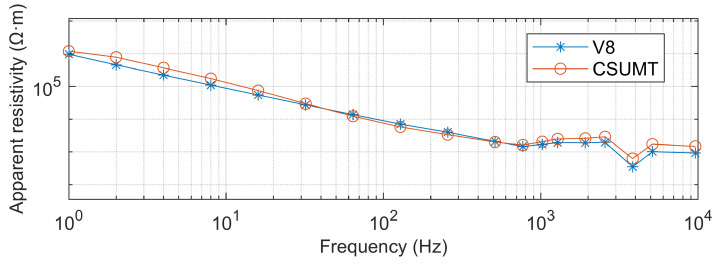
Comparison of apparent resistivity measurements between CSUMT and V8 receivers at the 1000 m point of the GY161 survey line.

The CSUMT and V8 receivers simultaneously receive signals from the transmitter. Figure 10, Figure 11 and Figure 12 show the electric field and magnetic field signals, as well as the measured apparent resistivity values, received by both receivers at the 1000 m point of the GY161 survey line. The electric field curves from both the CSUMT and V8 receivers are nearly identical. The magnetic field measurement curves from both receivers are also largely consistent, with slight deviations due to the slightly different positions of the magnetic sensors. The apparent resistivity values measured by the CSUMT and V8 receivers are also in close agreement.

**Figure 13:**
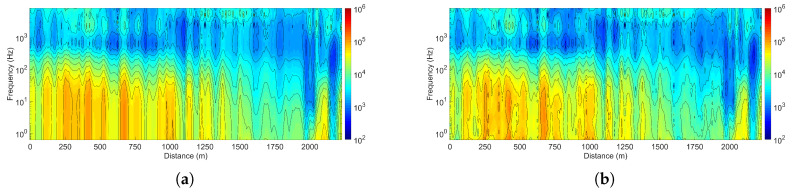
Comparison of apparent resistivity pseudo-sections along the GY161 survey line for the CSUMT and V8 receivers. (**a**) CSUMT receiver data. (**b**) V8 receiver data.

Figure 13 shows the pseudo-sections of apparent resistivity measurements along the GY161 survey line obtained by the CSUMT and V8 receivers. The results indicate a low-resistivity feature near the surface and predominantly high-resistivity characteristics at greater depths. At depths beneath the lateral positions around 2000 m and 2165 m along the survey line, low-resistivity anomalies are observed, which may suggest the presence of groundwater or metallic ore bodies. The apparent resistivity pseudo-sections obtained by the CSUMT and V8 receivers are largely consistent. The comparison experiment confirms that the CSUMT provides measurement results highly consistent with those of the V8, demonstrating its reliability for application in CSMT exploration.

**Figure 14:**
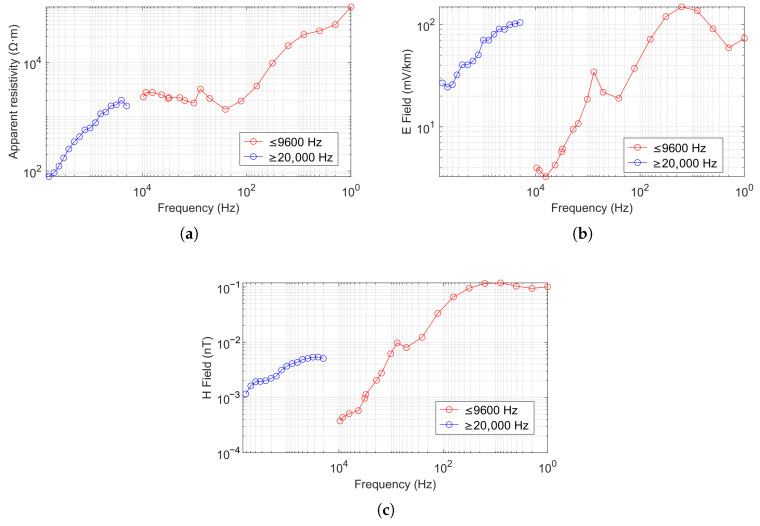
Full-band CSUMT measurement results at a specific location: (**a**) apparent resistivity, (**b**) electric field, and (**c**) magnetic field.

Since the Phoenix V8 system’s maximum frequency is 10 kHz, we did not use frequencies above 10 kHz for detection in the comparison experiment. However, to verify the wide frequency band characteristics of the CSUMT detector, we conducted measurements above 10 kHz independently using the CSUMT detector. The results for apparent resistivity, electric field, and magnetic field measurements are shown in Figure 14. It should be noted that the electric and magnetic field curves in Figure 14 show a noticeable amplitude gap between the low-frequency range (below 9600 Hz) and the high-frequency range (above 20 kHz). This discrepancy arises from the use of different transmission configurations: the low-frequency signals were generated by a high-power transmitter positioned approximately 6 km from the survey line, while the high-frequency signals were produced by a portable, low-power transmitter operated at shorter source-receiver distances near the measurement points. These differences in source current and geometry led to distinct field amplitudes but did not affect the accuracy of the apparent resistivity results.

## 4. Comparison Experiment Between the Inductive Magnetic Sensor and the SQUID Magnetic Sensor

### 4.1. Test Design and Field Work

**Figure 15:**
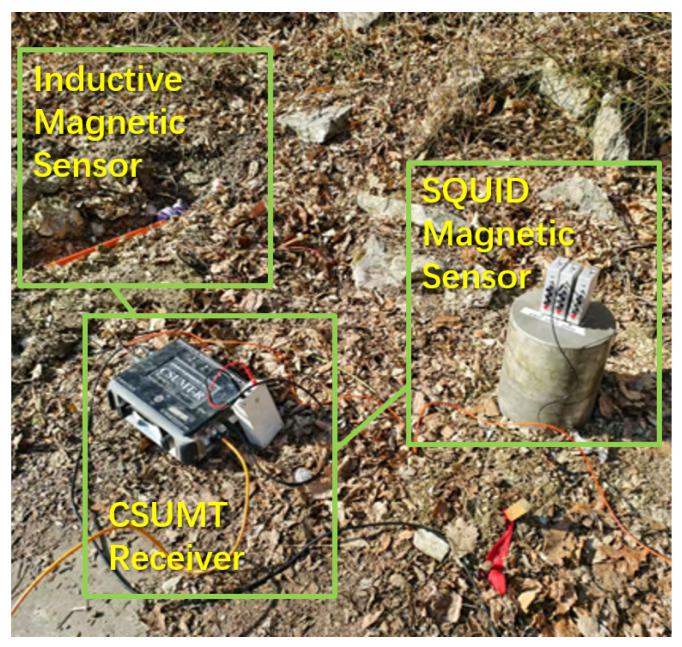
Photograph of the comparison experiment between SQUID magnetic sensor and inductive magnetic sensor.

To investigate the field applicability and performance characteristics of the SQUID magnetic sensor, a comparison experiment between the SQUID magnetic sensor and the inductive magnetic sensor was conducted in the Erlihe lead–zinc mining area of the Fengxian County ore concentration zone in Shaanxi Province. The locations of the transmitter and receiver are shown in Figure 5. The low-frequency transmission point is approximately 6 km from the survey line, with a transmission dipole length of 1 km, while the high-frequency transmission point is about 200 m from the survey line, with a dipole length of 24 m. A CSUMT receiver was used as the testing device for both sensors, with the SQUID magnetic sensor and the inductive magnetic sensor placed near the same measurement point and simultaneously connected to the same CSUMT receiver. As shown in Figure 15, the CSUMT receiver simultaneously records signals from both the SQUID and inductive magnetic sensors, allowing for a comparison of the measured magnetic field and apparent resistivity results.

### 4.2. Test Result

**Figure 16:**
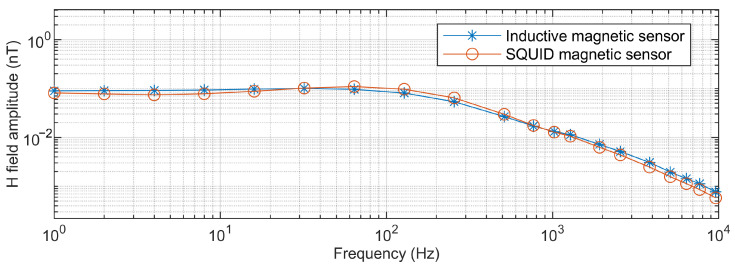
Comparison of magnetic field measurements by SQUID and inductive magnetic sensors at 1250 m along the GY161 survey line.

**Figure 17:**
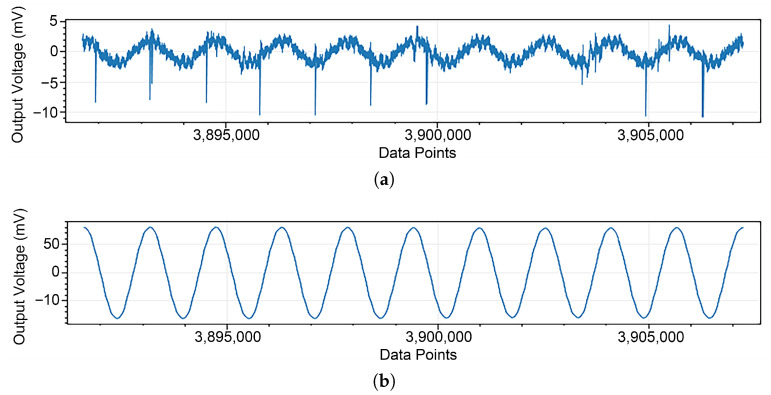
Time-domain waveforms of magnetic field measurements by SQUID and inductive magnetic sensors. (**a**) Signal waveform of the inductive magnetic sensor. (**b**) Signal waveform of the SQUID magnetic sensor.

Figure 16 shows a comparison of the magnetic field magnitudes measured using both the inductive magnetic sensor and the SQUID magnetic sensor at the 1250 m point along the GY161 survey line, with data collected simultaneously by a CSUMT receiver. The magnetic field measurements at different frequencies are generally consistent between the two sensors. During the measurement process, the SQUID sensor demonstrated the advantage of lower noise. As shown in Figure 17, the waveforms of the power-frequency magnetic field signals measured by the inductive magnetic sensor and the SQUID magnetic sensor are presented separately. The waveform recorded by the SQUID sensor exhibits lower noise and smoother signal characteristics. The signal stability is represented using the coefficient of variation:

(2) $$C_{v}=\frac{1}{\bar{x}}\sqrt{\frac{\sum_{i=1}^{n}{\left(x_{i}-\bar{x}\right)}^{2}}{n-1}}$$

**Figure 18:**
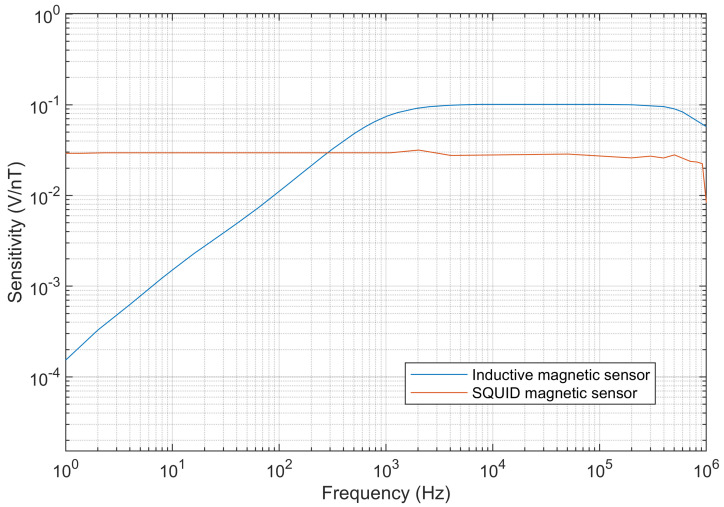
Comparison of sensitivity curves between SQUID and inductive magnetic sensors.

In the equation, $\bar{x}$ represents the mean of the data, *n* denotes the number of data points, and $x_{i}$ is each individual data point. Due to the lower measurement noise, the SQUID sensor yields more stable results. As shown in Table 3, the coefficient of variation of the SQUID magnetic sensor is smaller than that of the inductive magnetic sensor in most cases. Compared with the inductive magnetic sensor, the SQUID magnetic sensor has the advantage of maintaining a constant sensitivity across different frequencies. As illustrated in Figure 18, the sensitivity of the SQUID sensor remains nearly constant at 0.0295 V/nT within the frequency range of 1 Hz to 900 kHz, whereas the inductive magnetic sensor exhibits a flatter sensitivity range from 900 Hz to 750 kHz, with a rapid sensitivity drop outside this range as the frequency deviates. The consistent sensitivity of the SQUID sensor makes it suitable for applications across a broader frequency range, corresponding to a wider depth range in CSMT surveys.

## 5. Conclusions

This paper presents a detailed study of the self-developed Controlled-Source wide frequency range Magnetotelluric Detector (called CSUMT), and carries out systematic field experiments in the Fengxian County ore concentration zone in Shaanxi Province. The comparison experiments with the commercial V8 electromagnetic detection system show that the CSUMT detector achieves high consistency with the V8 system in measuring electric fields, magnetic fields, and apparent resistivity, validating its reliability and engineering applicability in complex geological environments.

In the magnetic sensor comparison experiment, the SQUID magnetic sensor mounted on the CSUMT detector demonstrates excellent performance in terms of measurement sensitivity, noise control, and data stability. It can stably detect magnetic field signals over a wider frequency range and with lower amplitudes, showcasing significant high-performance advantages. However, compared to inductive magnetic sensors, SQUID sensors have limitations in field deployment due to their reliance on low-temperature operation and higher costs. The inductive magnetic sensors, with their smaller size, lighter weight, and better environmental adaptability, remain a more practical option for rapid and extensive surveys in many cases.

Compared to previous studies on CSMT systems, our results highlight the superior frequency range and measurement stability of the CSUMT detector, as well as its potential for adapting to varied field conditions. However, this study is limited by the lack of comprehensive seismic validation data and long-term performance evaluations across diverse geological settings.

Future research should focus on extending the dataset to include controlled seismic testing and on refining the detector’s performance under varying environmental conditions. Further efforts are also needed to explore how to reduce the complexity and cost of SQUID-based magnetic sensors, making them more suitable for fast and large-scale surveys.

Overall, this study not only validates the practicality and advanced nature of the CSUMT detector but also provides important experimental data and practical references for the selection and integration of magnetic sensors in CSMT surveys. It also offers valuable insights for future technological improvements and for addressing the challenges faced in practical field applications.

## Figures and Tables

**Table 1:** Parameter of V8, Stratagem EH-5, GDP32II and CSUMT receiver.

Parameter	V8	Stratagem EH-5	GDP32II	CSUMT Receiver
Number of channels	6	5	up to 16	5
Frequency range	0.00005 Hz to 10 kHz	1 Hz to 96 kHz	0.0007 Hz to 8 kHz	1 Hz to 1 MHz
ADC	24-bit	32-bit	16-bit	24-bit
Size	21.5 × 23 × 14 cm	36 × 36 × 32 cm	43 × 41 × 23	34 × 28 × 15
Weight	7 kg	5.8 kg	13.7 kg to 19.1 kg	5.7 kg
Dynamic range	130 dB	127 dB	190 dB	135 dB @ 305 SPS

**Table 2:** Frequency schedule and duration of each measurement cycle.

Frequency (Hz)	Duration (s)
1	60
2	60
4	60
8	60
16	60
32	60
64	60
128	60
256	60
512	60
768	60
1024	60
1280	60
1920	60
2560	60
3840	60
5120	60
6400	60
7680	60
9600	60
15,800	18
20,013	18
25,128	18
31,588	18
39,766	18
50,155	18
63,015	18
79,277	18
100,721	18
126,680	18
159,584	18
201,442	18
256,000	18
323,368	18
396,387	18
491,520	18
614,400	18

**Table 3:** Coefficient of variation of data for inductive magnetic sensor and SQUID magnetic sensor.

Frequency (Hz)	Inductive Magnetic Sensor	SQUID Magnetic Sensor
1	0.06742	0.00197
2	0.00821	0.00049
4	0.00314	0.00022
8	0.00201	0.00042
16	0.00296	0.00335
32	0.00159	0.00095
64	0.00289	0.00316
128	0.00128	0.00121
256	0.00157	0.00154
512	0.00271	0.00248
768	0.00540	0.00710
1024	0.00485	0.00272

## Data Availability

Our research is supported by national projects; thus, the data are not publicly accessible due to a confidentiality agreement.
